# Mediterranean Diet and Health: Food Effects on Gut Microbiota and Disease Control

**DOI:** 10.3390/ijms150711678

**Published:** 2014-07-01

**Authors:** Federica Del Chierico, Pamela Vernocchi, Bruno Dallapiccola, Lorenza Putignani

**Affiliations:** 1 Unit of Metagenomics, Bambino Gesù Children’s Hospital, IRCCS, Piazza Sant’Onofrio, Rome 400165, Italy; E-Mails: federica.delchierico@opbg.net (F.D.C.); pamela.vernocchi@opbg.net (P.V.); 2 Interdepartmental Centre for Industrial Research-CIRI-AGRIFOOD, Alma Mater Studiorum, University of Bologna, Piazza Goidanich, Cesena-FC 47521, Italy; 3 Scientific Directorate, Bambino Gesù Children’s Hospital, IRCCS, Piazza Sant’Onofrio, Rome 400165, Italy; E-Mail: bruno.dallapiccola@opbg.net; 4 Unit of Parasitology, Bambino Gesù Children’s Hospital, IRCCS, Piazza Sant’Onofrio, Rome 400165, Italy

**Keywords:** Mediterranean diet (MD), Mediterranean diet patterns (MDP), nutritional algorithms, gut microbiota, food-related diseases, translational approaches, wellness therapy

## Abstract

The Mediterranean diet (MD) is considered one of the healthiest dietary models. Many of the characteristic components of the MD have functional features with positive effects on health and wellness. The MD adherence, calculated through various computational scores, can lead to a reduction of the incidence of major diseases (e.g., cancers, metabolic and cardiovascular syndromes, neurodegenerative diseases, type 2 diabetes and allergy). Furthermore, eating habits are the main significant determinants of the microbial multiplicity of the gut, and dietary components influence both microbial populations and their metabolic activities from the early stages of life. For this purpose, we present a study proposal relying on the generation of individual gut microbiota maps from MD-aware children/adolescents. The maps, based on meta-omics approaches, may be considered as new tools, acting as a systems biology-based proof of evidence to evaluate MD effects on gut microbiota homeostasis. Data integration of food metabotypes and gut microbiota “enterotypes” may allow one to interpret MD adherence and its effects on health in a new way, employable for the design of targeted diets and nutraceutical interventions in childcare and clinical management of food-related diseases, whose onset has been significantly shifted early in life.

## 1. Introduction

The Mediterranean diet (MD) is known to be one of the healthiest dietary habits [1]. Recently, the concept of dietary “pattern” has emerged as an alternative approach to examining the relationship between diet and the risk of developing chronic diseases [1]. Instead of looking at individual nutrients or foods, diet pattern analysis examines the effects of overall diet as a cluster outlook. From a speculative point of view, dietary patterns correspond to a broader concept of food and nutrient consumption and may thus be more predictive than individual foods or nutrients in establishing MD adherence and the impact on health. In fact, the beneficial effect of the MD is due to the synergic and interactive combinations of nutrients, rather than to isolated nutrients. Moreover, to evaluate the role of MD in wellness, the analysis of single nutrients may be insufficient to establish network information amongst foods and the effect of a single nutrient too little to be identified, hence requiring the evaluation of the cumulative effects of various nutrients in the context of dietary patterns [2].

**Figure 1:**
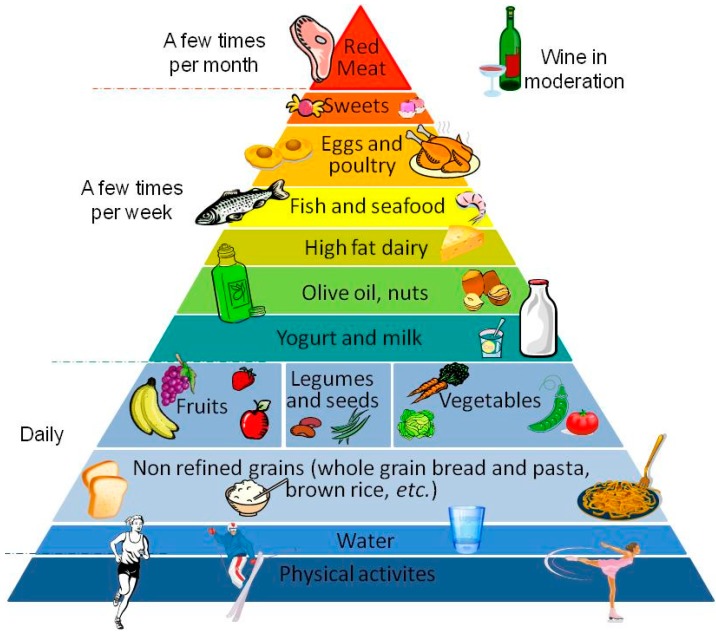
Pyramidal representation of Mediterranean dietary patterns (MDPs) and the frequency of recommended intake.

Mediterranean dietary patterns (MDPs) are characterized by the consumption of cereals (preferably as whole grains), legumes, nuts, vegetables and fruits, in high amount and frequency; MDPs also include reduced consumption of fish or seafood, white meat and eggs, moderate to small amounts of poultry and dairy products and low ethanol intake, usually in the form of wine. The principal source of dietary lipids of MDPs is olive oil, and an adequate daily intake of water should be guaranteed [3]. In addition, MDPs also include the practicing of physical activity in order to maintain a healthy physical and mental status [4] (Figure 1). On the contrary, the “Western” diet, diffused in industrialized countries, presents a higher intake of animal-derived foods (saturated fats), eggs, sweets, desserts and a lower eating of fruits, vegetables (fibers and micronutrients) and whole cereals [5]. Furthermore, in the MD, the consumption of traditional and local food products is a strength, respecting the seasonal availability and the biodiversity of food.

Many of the characteristic components of the MD have functional features, with positive effects on health and well-being; these may be responsible for the advantages associated with this diet [6]. Vegetables, fruits and nuts are the most important source of fibers and chemical compounds, like flavonoids, phytosterols, vitamins, terpenes and phenols, which give protection against oxidative processes, hence reducing the incidence of cardiovascular diseases (CVD) [6,7,8,9]. Furthermore, the consumption of olive oil, as the predominant fat intake, provides high oleic acid content and polyphenols, which have atherogenic, antioxidant and anti-inflammatory effects, reducing the cholesterol/high density lipoprotein (HDL) ratio and the concentration of the oxidized low density lipoprotein (LDL) [10,11,12]. Furthermore, olive oil has high levels of monounsaturated fatty acids (MUFAs) and a higher MUFA/saturated FAs (SFAs) ratio, which contribute to the protective effects [13,14]. Polyunsaturated FAs (PUFAs), contained in fish (*i.e*., eicosapentaenoic and docosahexaenoic acids), regulate hemostatic factors and provide protection against cardiac arrhythmias, cancer and hypertension and play a role in the preservation of cognitive functions [6,15,16]. Typically, MDPs include low-glycemic index and low-glycemic load foods, derived from the whole grain and other fiber-rich product intake, which have been associated with a lower risk of diabetes, mainly type 2, coronary health diseases (CHD) and cancer, while refined grain has been linked to the risk of diabetes, obesity, CHD and other chronic diseases [17,18]. The water-rich dairy products characteristic of the MD, such as yoghurt and cheese, are well tolerated by lactose-intolerant subjects. In addition, lactic acid bacteria, contained in the yoghurt, improve gastrointestinal (GI) health and immune response, displaying probiotic benefits [6]. Moreover, the consumption of yoghurt may induce positive modifications in the gut microbiota, which have been associated with a reduction of colon cancer risk indices [19].

Another important aspect in the MD, frequently ignored, is the low sodium intake; in fact, high sodium intake has been linked with high blood pressure, while consumption of salt-preserved foods has been connected with higher risk of stomach cancer, CHD and mortality [20]. Some of the beneficial effects of the MD in human diseases have been attributed to the polyphenols contained in red wine. Indeed, the antioxidant activity of these compounds may also be responsible for cytoprotective and cardioprotective action [21]. Particularly, MD adherence can be computed through various computational scores (e.g., the MD score for adults, the Mediterranean Diet Quality Index (KIDMED) for children [14] and Trichopoulou’s scale [22]), which are all based on dietary component quantities and types. For this purpose, questionnaires, including information on average consumption (never or rarely, times per month, week or day, as appropriate) of food items or beverages are administered. Portions can be estimated by “natural” units (e.g., one egg) or standard quantities (e.g., one teaspoon) and, when possible, supported by pictures of several foods and dishes, each representing a different portion, all taken under usual conditions. In addition, questionnaires include data on alcoholic beverage consumption for wine, beer, spirits and other drinks. For example, in Trichopoulou’s scale, a value of one and zero is assigned to each beneficial component (e.g., vegetable, legumes, fruits and nuts, cereal and fish) in the case of consumption or no-consumption, respectively. The inversion of the value is assigned to the so-called “detrimental” components (e.g., meat, poultry and dairy foods) and alcohol intake [22]. Based on the given answers, the test classifies the quality of the MD categorized as minimal, medium or maximal. However, the difficulty of the associations between dietary intake and disease cannot be ascribed to a single nutrient, but, rather, to multiple nutrients and foods. MDPs become then key parameters to explore the connections between nutrition and disease [23]. The new era of omics technologies are opening novel avenues to “nutrigenomics”, a field of genomics studying the effect of a specific food or diet on gene and expression profiles, leading to an advanced understanding of specific mechanisms between diet and host metabolism modulation [24]. MDPs and omics technologies may jointly provide an advanced original approach in nutraceuticals, merging eating behavior to systems medicine to identify benefits against chronic diseases.

## 2. MD and Diet Effects on Disease Risk

**Figure 2:**
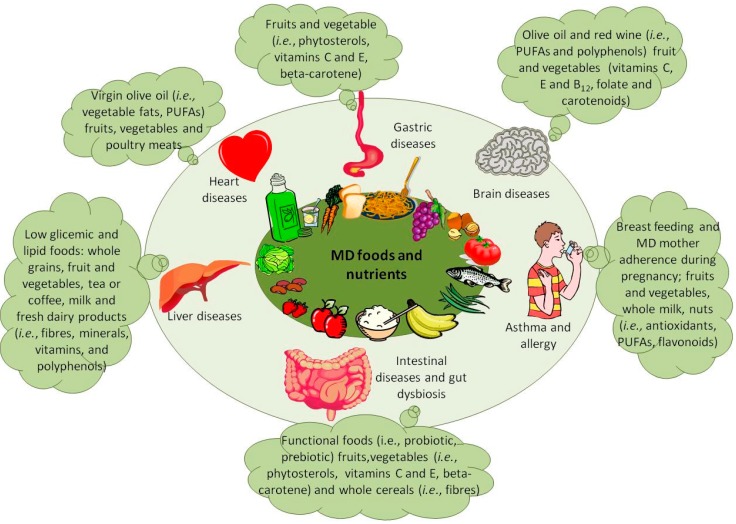
MD-related positive effects on diseases.

The high adherence to MD, as evidenced by studies on different groups of individuals, lead to a reduction of mortality and the incidence of major chronic diseases [23,25], such as cancer [26,27,28], metabolic and cardiovascular syndrome [29], neurodegenerative diseases [30], type 2 diabetes [31,32], fatty liver diseases [33] and allergy [34]. Moreover, MD is highly associated with an improved quality of life, which is translated into better psycho/physiological and metabolic profiles [35,36] (Figure 2). In recent years, dietary improvement has been related to a worldwide decrease of the gastric cancer rate [37]. For instance, in South Italy and in other countries of the Mediterranean basin (e.g., France and Greece), where the MD is widely diffused, compared to North Italy, the cancer mortality risk is generally lower than in other geographical regions, even if the full reasons for these decreases have not been completely unveiled [38].

Based on different studies, some MD components show reducing effects on gastric cancer occurrence [39,40,41]. Indeed, as reported by the second World Cancer Research Fund (WCRF) and American Institute for Cancer Research WCRF report (2007), non-starchy and allium vegetables, fruit and legumes probably protect against stomach cancer [40,42]. Olive oil and other vegetable fats, containing PUFAs, are reported to be inversely correlated to upper digestive, stomach and urinary tract cancer development risk [37]. On the contrary, low consumption of fruit and vegetables and a high intake of red meat, salty and smoked foods, grilled or barbecued meat increase the risk of gastric cancer development [39,40,41], while, fish, dairy products and alcohol consumption are less correlated with it [43,44].

Other cancers, such as breast cancer, whose etiology is multifactorial (e.g., genetics, behavior), seem to be associated with environmental agent exposition, such as diet. In fact, migrant studies have shown that, after moving from countries with a low breast cancer rate, an increased risk in one or two following generations is observed, confirming the importance of environmental factors [45]. On the other side, keeping traditional diet habits (e.g., rural) may decrease the development of breast cancer risk, because unhealthy food habits are often associated with the society’s evolution (e.g., acculturation) [46]. Some authors reported that vegetables, fruit, legumes, whole cereals, fish, poultry meat, soy and low-fat foods show an inverse relation with breast cancer development risk, while alcoholic intake is associated with a risk increase [47,48].

Moreover, specific food nutrients or micronutrients, belonging to principal MDPs, may play a role in breast cancer [49,50]. In fact, the intake of foods containing phytosterols, vitamins C and E, beta-carotene and calcium can exert, by the antioxidant effect on estrogen metabolic pathways, a protective action, including cell proliferation reduction [46]. Moreover, substances, such as ascorbic acid, carotenoids and other antioxidant vitamins, are inversely correlated with gastric cancer and neoplasms of the upper digestive and respiratory tract.

As is known, CVD risk prevention is implemented by the control of cardiometabolic diseases and metabolic syndromes, such as hypertension, hypercholesterolemia, glucose metabolism and obesity [29,51]. Specifically, MD micronutrients (e.g., polyphenols), contained in vegetables and fruit, have antioxidant effects and antiatherosclerotic properties protecting cells from the damage caused by reactive oxygen species (ROS)/reactive nitrogen species (RNS) [52,53]. Particularly, polyphenols, vitamins C and E, MUFAs, fibers and beta-carotene represent the key elements to improve the decrease of LDL cholesterol, triglycerides, fibrinogen and lipid oxidation indexes [54]. An important confirmation of the influence of diet on blood pressure was recently provided by Yokoyama *et al*. [55]. This meta-analysis of seven controlled clinical trials and 32 observational studies established a relationship between a vegetarian diet, which exclude or rarely include meats and a reduction of blood pressure, if compared with an omnivorous diet [55]. In CVD risk, other additional factors, called emotional disorders (e.g., anxiety and depression), aggravate or alter CVD status, counteracting the beneficial role of the MD habits [56]. Therefore, the high positive effects of the MD are limited to people with low levels of emotional disturbances. According to these evidences, behavioral and emotional disturbances of subjects with cardiovascular disorders should be always considered in planning appropriate dietary patterns [29].

Additionally, the MD has received remarkable attention for its role in the prevention of cognitive decline and the risk of dementia and Alzheimer’s disease (AD) [57]. First, dementia and cognitive decline have been related to various vascular risk factors [58], and the role of nutrition, and, especially, of the MD, has been widely studied in this context [2]. Indeed, many dietary components, such as vegetables, fruit, legumes and cereal, counteract oxidative stress and may promote beneficial effects on AD. Hence, the antioxidant compounds, contained in some of MDPs (e.g., polyphenols, vitamins C, E, B12, folate and carotenoids), may counteract the detrimental effects of oxidative stress in brain ageing and, thereby, reduce the AD risk [59]. Thus, the MD has been inversely associated with markers of oxidative stress [60] and lipid peroxidation [61]; during the last few years, some studies on New York and Chicago populations have detected that a lower AD risk may be actually associated with a higher MD adherence [62,63].

Furthermore, it has been demonstrated that subjects with high MD adherence have a reduction of 40% in brain infarcts and 10% in fatal and non-fatal CVD risk [58]. Because polyphenols and unsaturated FAs (UFAs) exert an anti-inflammatory action on the brain [64], they result in important to avoid inflammatory and neurodegenerative cascades, leading to AD and clinical dementia [65,66]. In the PREDIMED (prevención con dieta mediterránea) study of Valls-Pedret *et al.* [67], individuals with a high CVD risk have been enrolled and submitted to a dietary-intervention trial. The results showed that some components of the MD (*i.e.*, total olive oil, walnuts and one glass of wine), with antioxidant properties or rich in polyphenols, were independently associated with better cognitive function and high plasma levels of ω-3 FAs [68]. This evidence suggests that the association between MD adherence and cognitive functions may be mediated by vascular factors, but also by non-vascular biological mechanisms, such as oxidative stress, inflammation and metabolic disorders [69], supporting the importance of MD in health, aging and lifestyle [70].

In the last few years, some authors have indicated that MD adherence reduces the incidence of diabetes onset in Southern Europe [71], allowing a delayed anti-diabetes therapy by glycemic index control [72]. As previously reported, the “Western” dietary components are associated with the increase of obesity rates, leading to the type 2 diabetes increment. Hence, dietary habits and lifestyle both show a pivotal role in weight loss and, consequently, in diabetes prevention [73,74,75,76,77,78,79]. The MD principal protective compounds against diabetes are contained in fibers and vegetable fats; in particular, this protection is guaranteed by virgin olive oil intake (rich in MUFAs) used for cooking, spreading, dressing and frying foodstuffs [71,80]. In a cohort followed over 20 years, the intake of some single dietary constituent, such as whole grains, fruit, vegetables (especially leafy vegetables) [81,82], tea or coffee, nuts, milk and dairy products at a low-fat rate, was associated with the risk and the decreasing of the incidence (51%–52% and 25%, respectively) [83,84] of type 2 diabetes [73]. Hence, it seems that diets rich in MUFAs, such as the MD, improve insulin sensitivity, which allows better glycemic index and lipidic profile [85,86,87,88] control, with respect to ”Western”-like diets [86,89]. Interestingly, MD followers, as reported in some cohorts, show a potential protection from diabetes (e.g., lower inflammatory concentration markers in plasma), [90,91,92]. In a recent Italian study, 901 outpatients with type 2 diabetes who followed MD with high adherence showed a lower level of blood glucose (e.g., glycated hemoglobin HbA1c) and glucose after 2 h post-meal, independent of energy intake, age and lifestyle (e.g., physical activity) [74].

Current diet habits are based on the consumption of processed and manipulated foods, as well as foods consumed away from the place of origin [93,94], in contrast with “ancient” diets, based on foods consumed and sold locally, near the production sites [34,93,95]. Moreover, during the transition from an “ancient” to a modern diet, the intake of refined cereals, sugars and saturated fats has increased, as well as a deterioration of lifestyle habits, moving to sedentarism, obesity and fast food consumption [96,97]. Another widespread disturbance associated with diet is represented by allergic diseases (*i.e*., asthma, atopic allergy). The MD seems to be associated with asthma reduction; however, its effects are different and depend on the children’s age category (*i.e*., newborns, babies, school-aged and adolescents) [98,99].

Additionally, mother MD adherence during pregnancy may protect children from asthma and allergy in infancy [100]. Some authors have evidenced that breastfeeding prolonged until four months of life leads to a lower rate of asthma in infancy and has a positive effect on respiratory infections in children up to six years of age [101]. Moreover, it has also been shown that breastfed children are protected against allergic disease risks, even if this is not true when familial atopy already exists [102]. Some foods, such as fruits (e.g., apples, pears, citrus/kiwi fruit) [103], vegetables (e.g., tomatoes), whole milk [104], nuts [105], as well as nutrients rich in antioxidants, ω-3 PUFAs (e.g., fish oil) [106] and flavonoids [34,107], have a beneficial effect on asthma or wheezing.

However, geographical aspects must be taken into consideration; a higher asthma prevalence rate was registered in people who live in English-speaking countries, compared to people living in Mediterranean countries [100,108,109]. Currently, the strict MD adhesion of the mother during pregnancy and lactation represents the best prevention strategy to avoid allergy development in children who will be tomorrow’s adults. Therefore, these preventive actions during the prenatal period represent a critical stage to modulate the immune, respiratory and digestive system of the neonate, which might be otherwise affected for the rest of life.

## 3. MD Influences Gut Microbiota Composition

The holistic definition of “superorganism”, namely the sum of human and gut microbiota genomes, is the result of host genetic heritage and gut microbial ecological complexity [110]. When microbiota homeostasis is perturbed, dysbiosis leads to disease [111]. Food consumption produces thousands of products that, through digestion, induce direct (e.g., the uptake of calories, FAs, amino acids, carbohydrates) or indirect (microbial physiology) effects on host metabolism [112]. There is a few works in the literature that are related to the “integrated” interplay between diet, intestinal microbiota, genetics and mechanisms by which the MD drives the effects on gut microbiota and health [113]. In recent years, the role of the gut microbiota has achieved considerable significance in understanding human health and disease. Particularly, the alteration of microbiota and metabolism leads to dysbiosis, which represents the prelude to diseases, such as hepatic steatosis [114], metabolic syndromes [115], behavior abnormalities [116,117], metabolic disorders [118], inflammatory disorders [119,120], inflammatory bowel diseases (IBDs) [121] and minimal hepatic encephalopathy (MHE) [122,123]. The relationship between gut microbiota, health and disease had led to the use of probiotics, prebiotics and functional food, to prevent or treat some diseases, such as MHE [124,125], IBDs [126], inflammatory bowel syndrome [127,128], allergy [129], metabolic syndromes [130], hepatic steatosis [131] and colorectal cancer [132].

The main microbiome that is placed in our GI tract during adulthood is populated by approximately 10^14^ microbes, mainly consisting of bacteria. The largest part of the intestinal phylotypes belongs to a restricted set of phyla, such as Bacteroidetes, Firmicutes, Proteobacteria, Actinobacteria and Verrucomicrobia, but the relative amount of the bacterial phyla and species is usually altered in response to outside variables, especially diet. In fact, diet is one of the main significant determinants of the microbial multiplicity of the GI tract, and dietary components may be responsible for influencing both microbial populations and related distributions, from the early stages of life [133]. Therefore, even if each adult’s gut is shown to have a unique microbial community, with a stable structure (“core”) on a temporal scale of months, diet and other environmental factors actually affect microbiota metabolism, modulating resident (*i.e.*, autochthonous) and traveler (*i.e.*, allochthonous) microbes and causing the high dynamicity of the GI tract ecosystem [134]. In fact, as Zhang *et al*., [135] reported, dietary alterations are responsible for 57% of the gut microbiota’s entire variation, whereas genetic background explains only 12% [135]. De Filippo *et al.*, [136] compared the gut microbiota composition of European and rural African children, with respect to the diet contribution. This work demonstrated significant differences in gut microbiota between the two groups; in particular, African children showed a significant increase in Bacteroidetes and a decrease in Firmicutes, especially with the increment in *Prevotella* and *Xylanibacter* genera, containing gene sets for cellulose and xylan hydrolysis. Furthermore, Enterobacteriaceae were observed as decreased in African compared to European children. The authors hypothesized that gut microbiota co-evolves with the polysaccharide-rich diet of African individuals, allowing them to maximize energy intake from fibers and protecting them from inflammatory and gut diseases [136].

Recently, the analysis of gut microbial populations showed three main variants or “enterotypes” in adults represented by *Bacteroides*, *Prevotella* and *Ruminococcus* [137]. Wu *et al.* [43] investigated the relationship between dietary and environmental variables and gut microbiota in 98 healthy subjects, in a cross-sectional study approached by 16S rDNA pyrosequencing. The study showed that *Bacteroides* “enterotype” was highly associated with animal protein and saturated fat consumption, which implied that meat intake (e.g., as in the “Western” diet) actually characterized this “enterotype”. In contrast, the *Prevotella* “enterotype” was linked to high values of carbohydrates and simple sugars, indicating a relationship with a carbohydrate-based diet, typical of agrarian societies, and indeed, also vegetarians and vegans showed enrichment in the *Prevotella* “enterotype”. Furthermore, the authors performed a controlled-feeding trial based on a small subject cohort (10 subjects), which was randomized, subjected to high-fat/low-fiber or low-fat/high-fiber diets and sampled over 10 days. The results showed that microbiome profiles clearly changed within 24 h of diet, while the “enterotype” identity remained stable, indicating that long-term diet is strongly related with specific “enterotypes” [43]. Moreover, David and co-authors [138] studied, in six healthy volunteers, the effects of short-term diet on the inter-individual microbial gene expression. In particular, they administered two diets: the plant-based (*i.e*., rich in fruits and vegetables, legumes and grains) and the animal-based diet (*i.e.*, meats, cheeses and eggs). The latter increased the total count of bile-tolerant microorganisms (e.g., *Alistipes* spp*.*, *Bilophila* spp*.* and *Bacteroides* spp.) and decreased the levels of Firmicutes able to metabolize plant polysaccharides (e.g., *Roseburia* spp., *Eubacterium rectale* and *Ruminococcus bromii*). Correlation analysis of short chain fatty acid (SCFA) fecal content and bacterial clusters suggested that macronutrients changed the microbial metabolic activity. When the authors correlated SCFA concentrations with the abundance of bacterial clusters, they found significant positive relationships between clusters composed of putrefactive microbes (e.g., *Alistipes putredinis* and *Bacteroides* spp.) and SCFAs, terminal products of amino acid fermentation. The authors also detected important positive correlations between clusters of saccharolytic bacteria (e.g., *Roseburia*, *E. rectale* and *Faecalibacterium prausnitzii*) and carbohydrate fermentation yields. The animal-based diet was linked with augmented expression of: (i) vitamin biosynthesis genes; (ii) degradation of polycyclic aromatic hydrocarbons (*i.e.*, carcinogenic compounds produced from the meat burning); and (iii) β-lactamase genes. Remarkably, the plant- and animal-based diets also promoted transcriptionally-specific responses with a divergence in gene profile abundance in herbivorous and carnivorous mammal gut microbiota, as well as transitions from amino acid catabolism to biosynthesis. Finally, augmentations in the abundance and activity of *Bilophila wadsworthia* in the gut microbiota of the animal-based diet subjects support a relationship between fat intake, bile acids and the presence of microorganisms capable of activating inflammatory bowel disease [139]. Indeed, to increase the knowledge on the microbial community adaptation to diet, fecal DNA from 33 mammalian species and 18 humans, with detailed diet records, was investigated by 16S rDNA targeted-metagenomics. The alterations of the microbiota due to diet were comparable across different mammalian species. Complete catalogs of microbiome genes (e.g., encoding carbohydrate-active enzymes and proteases) were predicted from bacterial species groups. These results demonstrated the value of characterizing vertebrate gut microbiomes to comprehend host evolutionary records [139]. Walker *et al*., [140] studied the influence of specifically controlled diets in 14 obese men. To the study subjects, three type of diets ((i) high in resistant starch; (ii) non-starch polysaccharides; and (iii) a reduced carbohydrate weight loss diet) were administered over 10 weeks. Three hundred and twenty microbial phylotypes were detected in fecal samples by the analysis of 16S rDNA sequences. In subjects alimented by the resistant starch diet, relatives of *Ruminococcus bromii* increased up to 17% of total bacteria, while in the non-starch polysaccharides diet, they accounted for 3.8%; whereas the *Oscillibacter* group increased in the resistant starch and weight loss diets. Furthermore, on the resistant starch diet, relatives of *E. rectale* increased to 10.1%, but decreased, along with *Collinsella aerofaciens*, on the weight loss diet [140].

The interaction involving diet and the microbiota has been associated wtih the rising frequency of chronic diseases linked with the “Western” lifestyle. The study of Siddharth *et al*., [141] investigated germ-free mice transplanted by human fecal, afterwards fed with the “Western” and normal diet. The study also included a diet crossover. The gut composition of “Western” diet and normal chow mice was different. For example, *Marvinbryantia* was significantly co-correlated to *Lachnospiraceae incertae sedis* and *Alistipes* to *Bacteroides* under “Western” diet, if compared to normal chow. Conversely, *Clostridium* XVIII was co-correlated with *Coprobacillus* under a normal chow diet. These data displayed that diet strongly influences the bacterial ecology. Moreover, in this study, healthy human subjects were fed in clinical settings using a high-fat/low-fiber or a low-fat/high-fiber diet, respectively. The results showed that the abundance of *Bacteroides* were significantly associated with the “Western” diet. Conversely, *Blautia*, *Flavonifractor* and *Butyricicoccus* were significantly higher in the low-fat diet subjects. Furthermore, the authors investigated the gut microbiota composition across geography by comparing three populations ((i) Western people in the USA; (ii) Guahibo Amerindians residing in Venezuela; and (iii) rural communities in Malawi) to establish the *Bacteroides* abundance connection with the “Western” diet and the diverse geography of subjects [142]. In this study, 531 individuals (e.g., healthy adults and children), their geographical location and dietary habits were examined. Particularly, in the Malawian population, characterized by a low-fat/high-fiber diet, the abundance of the *Bacteroides* was lowest, but, between the Venezuelan and USA populations, no significant differences were found [142].

Weight loss diets (e.g., a high intake of protein, but a low intake of fermentable carbohydrate) may alter microbial activity and bacterial inhabitants in the large intestine. In the study of Duncan *et al*. [143], 19 obese healthy subjects were submitted to three different diet intakes: (i) maintenance for three days (399 g carbohydrate/day); (ii) high protein/medium carbohydrate (164 g/day); and (iii) high protein/low carbohydrate (24 g/day); each for four weeks. At the end of each dietary regimen, fecal samples were analyzed by capillary gas chromatography (GC) and fluorescence *in situ* hybridization (FISH) analysis. The results showed that SCFAs, with a disproportionate butyrate reduction, decreased with the decreasing of the carbohydrates diets. For the bacteria population, no noteworthy modification was observed in the relative counts of the *Bacteroides* or *Clostridium* XIVa, IX or IV clusters. On the contrary, the *Roseburia* spp., the *E. rectale* subgroup and the *Bifidobacteria* decreased with the carbohydrate decreasing. Thereafter, the reduction of butyrate-producing bacteria, *Roseburia* spp. and *E. rectale*, was correlated with the decreasing of fecal butyrate. The changes in fecal butyrate provided the strongest evidence that its production is largely due to the diet content of fermentable carbohydrate, and the proportions of specific colonic bacteria groups respond to dietary carbohydrate intake [143].

Marlow *et al*. [144] conducted a pilot study using *C*-reactive protein (CRP) [145] and a micronucleus assay [146] as inflammation biomarkers and gut microbiota transcriptomics to test the ability of the MD to reduce inflammation in Crohn’s disease. For this purpose, the authors added foods that previous research showed to be beneficial in reducing inflammation and removed foods that were shown to be detrimental to Crohn’s disease patients [147]. The results showed that consuming the MD resulted in a small reduction of the inflammation biomarker levels, also influencing gut microbiota, increasing Bacteroidetes and *Clostridium* clusters and decreasing the Proteobacteria and Bacillaceae population [144].

## 4. Conclusions and Future Perspectives

The MD’s healthy impact on childhood health and disease prevention is only presumed on the basis of the current opinion and little scientific evidence. Currently, only the NU-AGE project (“New dietary strategies addressing the specific needs of elderly population for an healthy aging in Europe”), by the use of omics techniques, has the goal of linking the dietary effect to cellular inflammation in the elderly [148]. Up to now, literature concerning the MD’s role during childhood is still lacking. For this reason, our proposal is to conceive of a dedicated conceptual framework and a systems biology-based project design to evaluate the MD’s effect on gut microbiota, by recruiting healthy young individuals grouped into three age stages: (i) 6–11 years, childhood; (ii) 12–16 years, puberty; and (iii) 17–21 years, adolescence.

**Figure 3:**
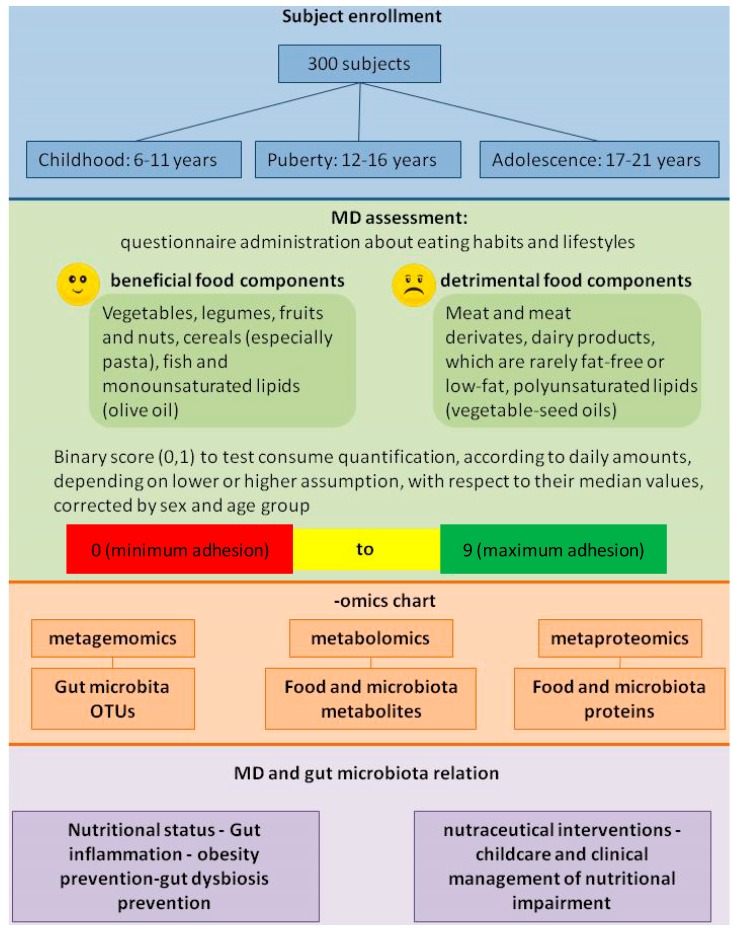
MD and gut microbiota relationship: A proposal for a study design.

Trichopoulou’s scale (0–9 MFP scores) will be used to quantify MD adhesion. The gut microbiota-MD relationship will be investigated by metagenomics, metabolomics and metaproteomics on fecal samples. Based on computed scores, individuals will be categorized into MDP-related subsets, and “nutrimetabolomics” tools will describe food metabolism products for the entire group of enrolled individuals. Gut metabolism outlines will be associated with age range subsets in order to infer direct effects on the host’s health (e.g., food metabolism) during growth until adulthood. Then, the meta-omics maps will be integrated in order to describe the relationship between metabotypes and “enterotypes” in an age-dependent manner, by stratifying individual MDPs (Figure 3).

Within this *scenario*, the assessment of a scientific proof of evidence, on the correlation between MD adherence and food-derived metabotypes and “enterotypes”, could allow one to develop targeted diets, nutraceutical interventions and improve the childcare and clinical management of people with nutritional impairment, from childhood. Hence, the description of MDPs and related alterations of gut microbiota profiling will be used to prevent, by the most innovative translational approaches of systems medicine, food-related diseases (e.g., obesity, metabolic syndrome, diabetes, *etc*.), whose onset has been significantly shifted to early in life. The next challenge resides in the new “vision” of the gut microbiota considered as a multifunctional “organ” constantly subjected to food-driven perturbation.
